# Supplementary material for: Bacillus halophilus BH-8 Combined with Coal Gangue as a Composite Microbial Agent for the Rehabilitation of Saline-Alkali Land
Source: Microorganisms. 2025 Feb 27;13(3):532. doi: 10.3390/microorganisms13030532 (PMC11945998; doi:10.3390/microorganisms13030532)
Supplement: Supplementary file 1 [file microorganisms-13-00532-s001.zip › microorganisms-3454691-supplementary-proof done.pdf]

Supplementary Material

Supplementary Figures

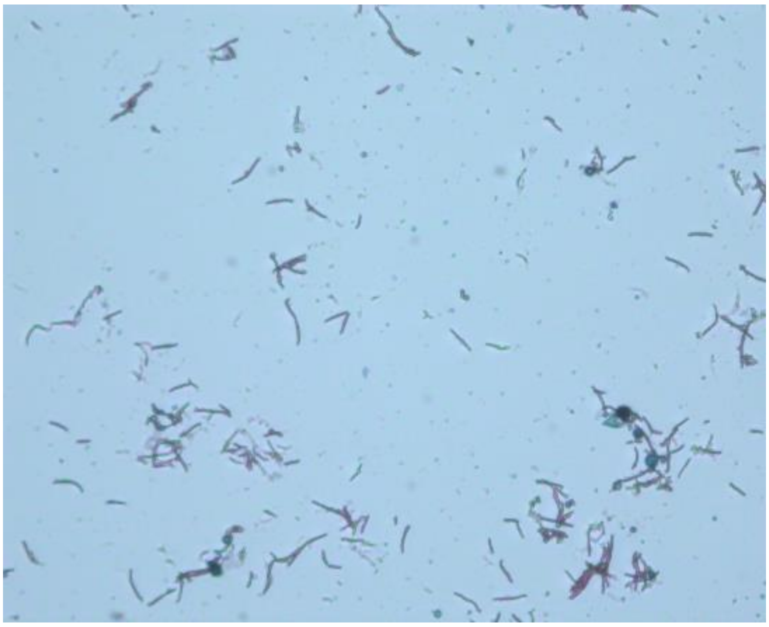

Figure S1 Inverted fluorescence microscope analysis of sporulation capability in *Bacillus halophilus* BH-8. Blue-green indicates typical spore structures, while red represents vegetative cells.

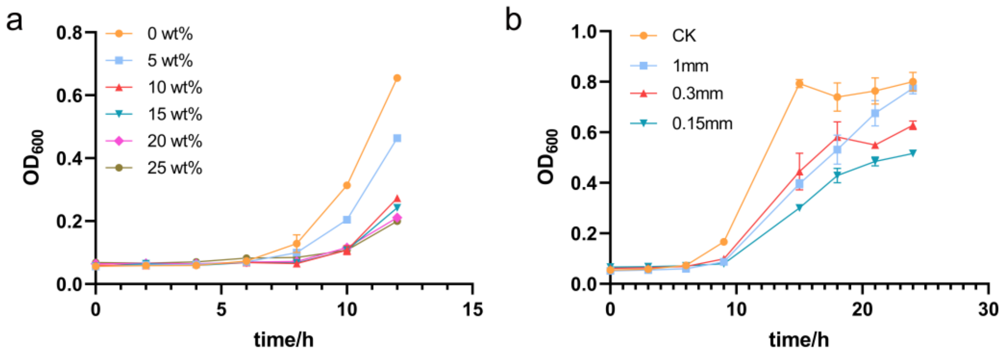

Figure S2 The growth of BH-8 co-cultured with coal gangue at varied concentrations and particle sizes. (a) Growth of BH-8 strain with indicated concentrations of coal gangue supplementation. (b) Growth curve of BH-8 strain with indicated particle sizes of coal gangue supplementation.

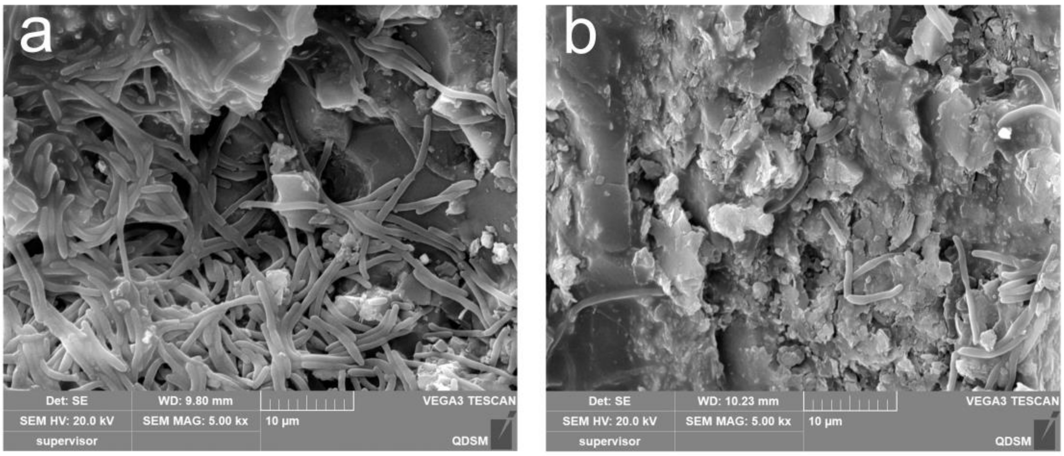

Figure S3 SEM images of BH-8 attached to coal gangue. (a) The surface morphology of coal gangue with a particle size of 1 mm and the attachment of BH-8. (b) The morphology of coal gangue with a particle size of 0.15 mm and the attachment of BH-8.

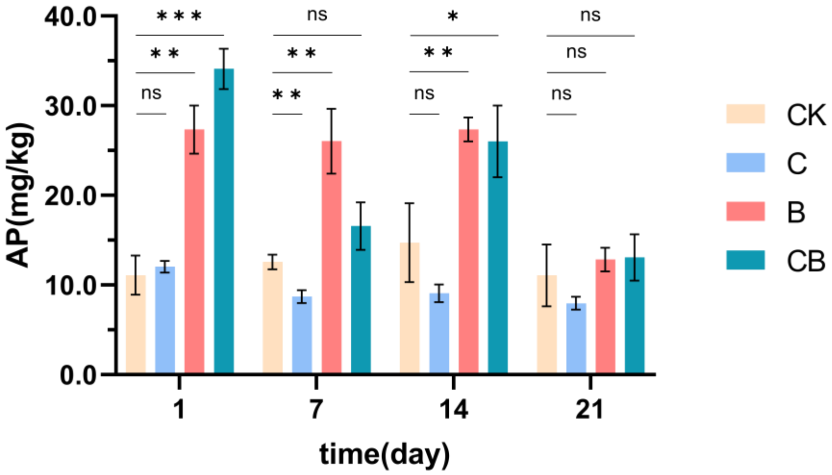

Figure S4 Changes in soil available phosphorus (AP) content

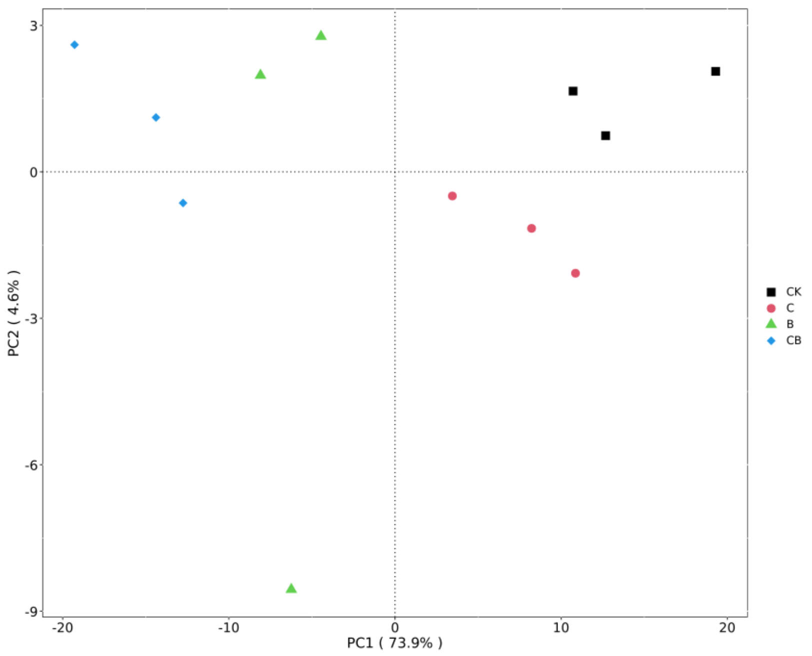

Figure S5 Principal component analysis (PCA) based on the phylum level.

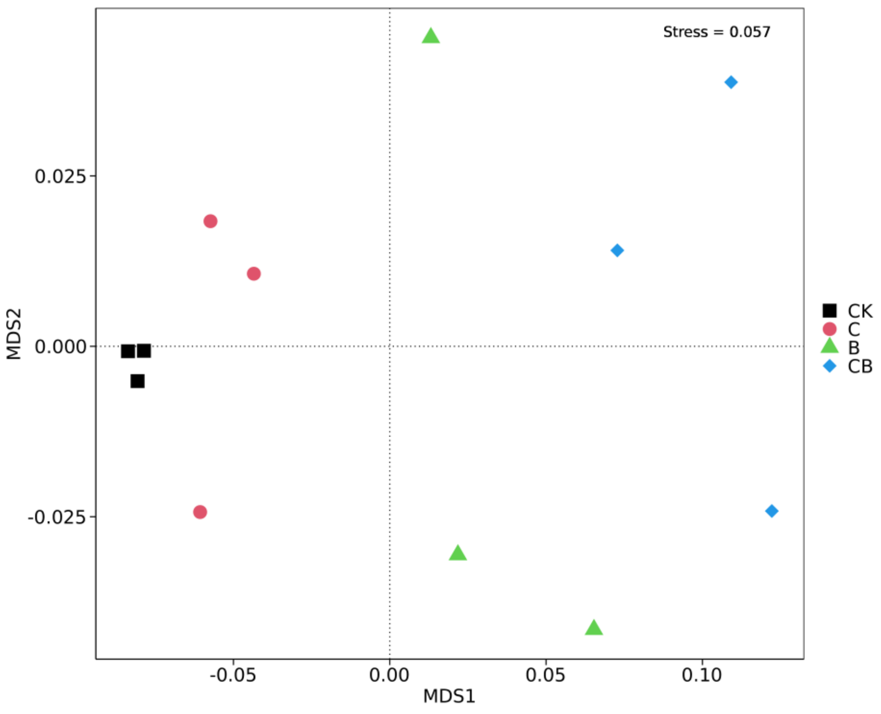

Figure S6 Non-metric multidimensional scaling (NMDS) based on the phylum level.

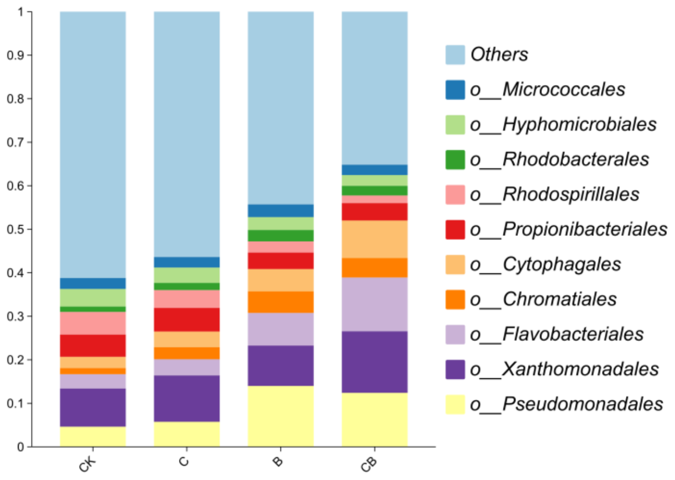

Figure S7 Histogram of relative species abundance at the Order level.

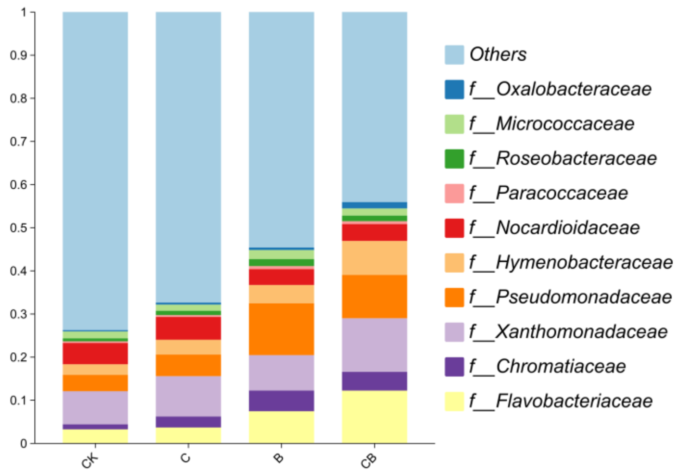

Figure S8 Histogram of relative species abundance at the Family level.

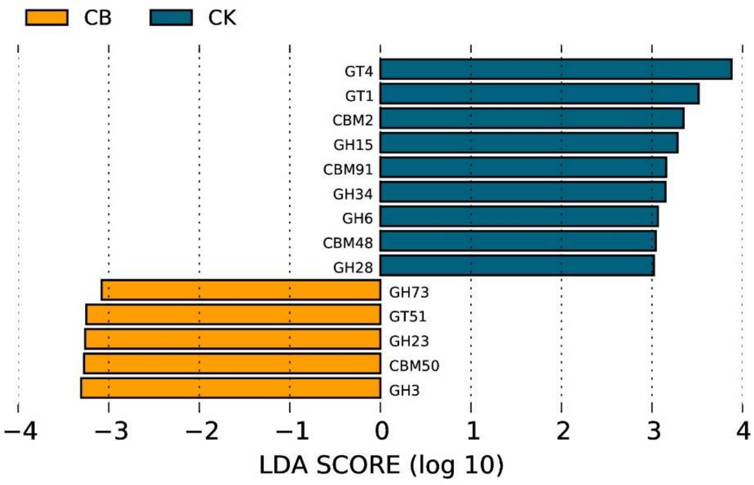

Figure S9 Linear discriminant analysis (LDA) of CAZy Database.

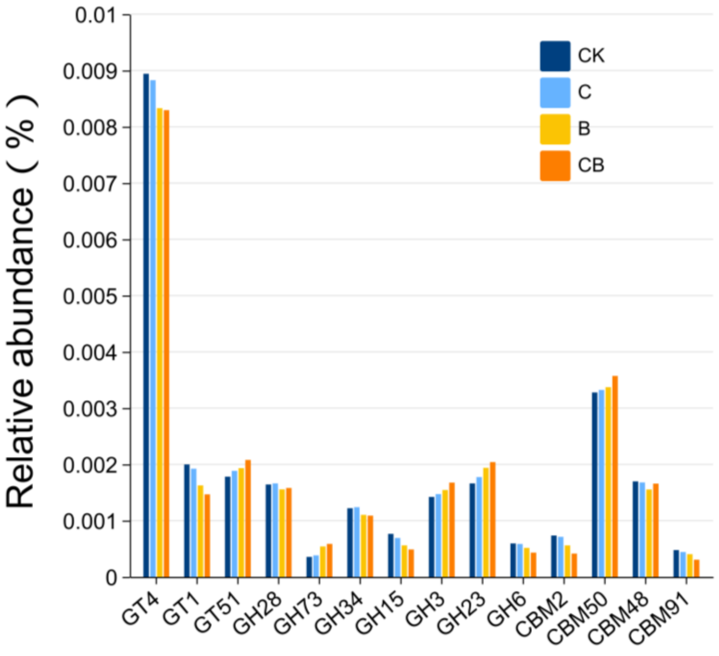

Figure S10 The histogram of relative functional abundance

Supplementary Tables

Table S1 Biochemical and enzymatic characteristics determination of BH-8

Experiment Name	Result
VP	-
Indole	-
H_2S	+
Nitrate Reduction	-
Citrate Utilization	-
Starch Hydrolysis	+
catalase	+
Urease	-
Oxidase	+

Table S2 Chemical Composition of Coal Gangue

ingredients	SiO_2	Al_2O_3	MgO	Fe_2O_3	K_2O	K_2O	CaO	TiO_2	Na_2O
Content (%)	56.844	22.421	2.132	9.048	3.847	3.847	2.261	1.092	1.464

Table S3 Metal Element Content of Coal Gangue

ingredients	Mn	Ga	Rb	Zn	Sr	Zr	Nb	Ba
Content (%)	0.129	0.027	0.053	0.037	0.172	0.094	0.011	0.083

Table S4 Gene function annotation

Class	
CBM2	Carbohydrate Binding Module Family 2. CBM2 modules generally comprise 90 to 100 residues that adopt a canonical beta-jelly-roll fold.
CBM48	Carbohydrate Binding Module Family 48. Family CBM48 contains modules able to bind various linear and cyclic α-glucans related to and derived from starch and glycogen.
CBM50	Carbohydrate Binding Module Family 50. CBM50 modules are about 50 amino acids long and its members are also known as LysM domains. They bind to the N-acetylglucosamine residues in bacterial peptidoglycans and in chitin.
CBM91	Binding to xylans
GT1	UDP-glucuronosyltransferase;

	zeatin O-beta-xylosyltransferase;<br>2-hydroxyacylsphingosine 1-beta-galactosyltransferase;
	N-acylsphingosine galactosyltransferase;
	flavonol 3-O-glucosyltransferase;
	anthocyanidin 3-O-glucosyltransferase;
	sinapate 1-glucosyltransferase;
	indole-3-acetate beta-glucosyltransferase;
	flavonol L-rhamnosyltransferase;
	sterol glucosyltransferase;
	UDP-Glc: 4-hydroxybenzoate 4-O-beta-glucosyltransferase;
	zeatin O-beta-glucosyltransferase;
	limonoid glucosyltransferase;
	UDP-GlcA: baicalein 7-O-beta-glucuronosyltransferase;
	UDP-Glc: chalcone 4'-O-beta-glucosyltransferase;
	ecdysteroid UDP-glucosyltransferase;
	salicylic acid beta-glucosyltransferase;
	anthocyanin 3-O-galactosyltransferase;
	anthocyanin 5-O-glucosyltransferase;
	dTDP-beta-2-deoxy-L-fucose: alpha-L-2-deoxyfucosyltransferase;
	UDP-beta-L-rhamnose: alpha-L-rhamnosyltransferase;
	zeaxanthin glucosyltransferase;
	UDP-Glc: flavone-6-C-glucosytransferase;
	UDP-Glc: hydroxycinnamic acid O-beta-glucosyltransferase;
	UDP-Glc: cinnamate beta-glucosyltransferase;<br>UDP-Glc: cinnamoyl O-beta-glucosyltransferase;<br>UDP-Arap: flavone-C-arabinosytransferase;
	[inverting] UDP-Glc: ginsenoside 3-O-glucosyltransferase;
	[inverting] UDP-Glc: 3-O-glucosyl-protopanaxadiol-type ginsenoside<br>2''-O-glucosyltransferase;
	UDP-Glc: p-hydroxymandelonitrile-O-glucosyltransferase
GT4	sucrose synthase;
	GDP-Man: Man3GlcNAc2-PP-dolichol/Man4GlcNAc2-PP-dolichol<br>alpha-1,2-mannosyltransferase;
	GDP-Man: Man1GlcNAc2-PP-dolichol alpha-1,3-mannosyltransferase;
	phosphatidylinositol alpha-mannosyltransferase;
	lipopolysaccharide N-acetylglucosaminyltransferase;
	alpha-glucosyltransferase;
	sucrose-phosphate synthase;
	digalactosyldiacylglycerol synthase;
	1,2-diacylglycerol 3-glucosyltransferase;
	diglucosyl diacylglycerol synthase;
	trehalose phosphorylase;
	NDP-Glc: alpha-glucose alpha-glucosyltransferase / alpha,alpha-trehalose

	synthase;
	GDP-Man: Man2GlcNAc2-PP-dolichol alpha-1,6-mannosyltransferase;
	UDP-GlcNAc: 2-deoxystreptamine alpha-N-acetylglucosaminyltransferase;
	UDP-GlcNAc: ribostamycin alpha-N-acetylglucosaminyltransferase;
	UDP-Gal alpha-galactosyltransferase;
	UDP-Xyl alpha-xylosyltransferase;
	UDP-GlcA alpha-glucuronyltransferase;
	UDP-Glc alpha-glucosyltransferase;
	UDP-GalNAc: GalNAc-PP-Und alpha-1,3-N-acetylgalactosaminyltransferase;
	UDP-GalNAc: N, N'-diacetylbacillosaminyl-PP-Und alpha-1,3-N-acetylgalactosaminyltransferase;
	ADP-dependent alpha-maltose-1-phosphate synthase;
	UDP-GlcNAc: polypeptide alpha-N-acetylglucosaminyltransferase;
	GDP-Man: alpha-1,4-mannosyltransferase;
	UDP-GlcNAc: alpha-N-acetylglucosaminyltransferase;
GT51	murein polymerase
GH3	beta-glucosidase;
	xylan 1,4-beta-xylosidase;
	beta-glucosylceramidase;
	beta-N-acetylhexosaminidase;
	glucan 1,4-beta-glucosidase;
	alpha-L-arabinofuranosidase;
	isoprimeverose-producing oligoxyloglucan hydrolase;
	exo-1,3-1,4-glucanase;
	beta-N-acetylglucosaminide phosphorylases;
	coniferin beta-glucosidase;
	beta-1,2-glucosidase;
	beta-1,3-glucosidase;
	xyloglucan-specific exo-beta-1,4-glucanase / exo-xyloglucanase;
	stevioside-beta-1,2-glucosidase;
	lichenase / endo-beta-1,3-1,4-glucanase;
	protodioscin 26-O-beta-D-glucosidase;
	beta-glucuronidase;
	avenacinase;
	tomatinase beta-1,2-glucosidase
GH6	endoglucanase;
	cellobiohydrolase;
	lichenase / endo-beta-1,3-1,4-glucanase;
GH15	beta-1,3-glucanase
GH23	lysozyme type G;
	peptidoglycan lyase; chitinase

GH28	polygalacturonase;
	exo-polygalacturonase;
	exo-polygalacturonosidase;
	rhamnogalacturonase;
	rhamnogalacturonan alpha-1,2-galacturonohydrolase;
	xylogalacturonan hydrolase
GH34	sialidase or neuraminidase
GH73	lysozyme;
	mannosyl-glycoprotein endo-beta-N-acetylglucosaminidase;
	peptidoglycan hydrolase
